# DETECTION OF R576 INTERLEUKIN-4 RECEPTOR AN ALLELE GENE, SERUM INTERLEUKIN-4, AND EOSINOPHILIC CATIONIC PROTEIN IN ATOPIC DERMATITIS PATIENTS

**DOI:** 10.4103/0019-5154.48983

**Published:** 2009

**Authors:** M Y Abdel-Mawla, Y Mostafa, Y Abuel-Majd, Rasha Attwa

**Affiliations:** *From the Department of Dermatology, Zagazig Faculty of Medicine, Zagazig, Egypt*; 1 *From the Department of Medical Biochemistry, Zagazig Faculty of Medicine, Zagazig, Egypt*

**Keywords:** *Allergy*, *asthma*, *atopy*, *cell*, *dermatitis*, *eosinophils*, *interleukin*, *mast*, *patients*, *proteins*, *remodeling*

## Abstract

Atopic dermatitis (AD) is a chronic pruritic skin disease. It results from a complex interplay between strong genetic and environmental factors. The aim of this work was to study some biochemical markers of the dermatosis. This included detection of R576 interleukin-4 receptor alpha allele gene. Twenty five patients with AD and 25 controls participated in this study.

## Introduction

Atopic dermatitis (AD) is recognized as a strongly heritable chronic, pruritic inflammatory skin condition that is most common in early childhood and predominantly affects the skin flexures. Current evidence indicates that AD is strongly genetic, with enhanced levels of phenotype concordance reported in monozygotic relative to dizygotic twins.[1] The individual genetic factors or genes that contribute to the trait's cause are relative to other complex genetic diseases proving amenable to identification.[1] The concept of the atopic march refers to the sequential development of AD, allergic rhinitis, and asthma. Longitudinal studies reveal that AD is a risk factor for the future development of allergic airway disease.[2] Multiple genetic loci are associated with various phenotypes of atopic disease, and three genetic loci are associated with both AD and asthma (5q31-33, 11q13, and 13q12-14). In AD, allergen-induced skin lesions display two phases – an initial phase with predominantly interleukin (IL)-4-producing T helper (Th)2 cells and a subsequent phase after 24–48 hours characterized by IFNγ-producing Th1 cells.[3] This switch is thought to be initiated by the local production of IL-12 from infiltrating eosinophils.[4] Activated T cells expressing Fas ligand have also been shown to induce keratinocyte apoptosis contributing to the spongiosis found in acute AD.[5] This process is mediated by Interferon (IFN)γ, which upregulates Fas on keratinocytes.[6]

### Aim

Aim of this study was to identify some immunological and chemical markers in AD and their relation to disease severity. Aim was also to detect genotype R576 IL-4 receptor α allele and to clarify its segregation with AD as well as its usefulness as clinical marker of the disease.

## Materials and Methods

Twenty five patients with AD and 25 age and sex matched controls participated in this study. All the participants had received no antihistamines or systemic or topical corticosteroids during the period of three weeks before clinical evaluation (a wash-out period), and were subjected to skin-prick test. AD diagnosis was based on the criteria of Hanifin and Rajka.[7] The severity of AD was measured according to Emerson *et al,* by using the Nottingham eczema severity score.[8]

### Control group

Twenty five healthy, nonatopic, age and sex matched, and unrelated volunteers comprised the control group. They were enrolled in the study if their skin testing were negative and after excluding history of allergic conditions.

All subjects included in this study were subjected to the following: a complete clinical history was obtained followed by clinical examination. Stool and urine analysis was done to exclude parasitic infestations for its affect on eosinophil count and activity.[9] Determination of the presence of specific IgE to certain allergens was conducted by skin-prick test according to Tipton.[10] Genotyping for R576 IL-4 receptor α allele was assessed by PCR-based restriction fragment length polymorphism according to Rosa-Rosa *et al*.[11] Serum IL-4 level was estimated by ELISA according to Banchereau.[12] Determination of serum eosinophil cationic protein (ECP) level was done by chemoluminescence method according to D Amato and Liccard.[13] Also, complete blood analysis was done to determine the eosinophil count in peripheral blood.[14] Serum total IgE level was estimated by ELISA according to Dorrington and Bennich.[15]

Determination of R576 IL-4 α allele was by PCR-based restriction fragment length polymorphism.

### DNA extraction

DNA was isolated using the PUREGENE DNA isolation kit purchased from Gentra. DNA was extracted according to the method of Bubbon.[16]

### Statistical evaluation

The statistical method used for analysis of the data was according to Kirkwood.[17] The statistical data were calculated for mean standard deviation (SD), standard error (SE), student's *t*-test, analysis 2 of variance (*F*-test), correlation coefficient, Chi-square (X^2^) test, exact Fisher exact, and Odds ratio and relative risk.

## Results

The results of this study showed that the most common allergens causing positive skin test among atopic patients were mixed pollens (46.6%), hay dust (33.3%), smoke (21.3%), house dust mite (18.6%) and mixed fungus (16%), cotton and wool (9.3%), mixed feather (6.6%), and animal dander (5.3%).

There was a statistically significant association between R576 allele and atopy as compared with control group (*P* < 0.001). There was also a significant association between homozygosity for the R/R576 allele and atopy (*P =* 0.02). The relative risk of R576 allele in atopy was 7.3. For homozygous R/R576, there was a statistical significance for the severe versus mild disease (*P* = 0.03) [Table 1]. Levels of total IgE, IL-4, eosinophil count, and ECP in sera of patients were found to be significantly increased in comparison of their levels in the control samples [Table 2]. There were also significant correlation between levels of total IgE, ECP, and total eosinophil count with clinical scoring, but there was no significant correlation between serum IL-4 levels and severity of AD. There was a highly significant increase in the total IgE, eosinophil count, and ECP levels in homozygous R576/R576 atopic patients (*P* < 0.001). Also, there was no statistically significant difference between the serum IL-4 values and allelic variants in all atopic patients. There was a significant positive correlation between serum ECP and peripheral blood eosinophil cell count, and serum total IgE. There was no statistically significant correlation between serum IL-4 and serum ECP, peripheral blood eosinophils cell count, or total serum IgE (see tables).

**Table 1:** Effect of R576 and Q576 IL-4Rα allelic variant on atopic dermatitis severity

Dermatitis severity	Q576/Q576 (%)	Q576/R576 (%)	R576/R576 (%)
Mild* Moderate** Severe***	5 (62.5) 3 (37.5) 0 (0)	5 (41.7) 5 (41.7) 2 (16.7)	0 (0) 2 (40) 3 (60)
*P* value: severe vs. mild	0.01	0.20	0.03

* Nottingham eczema severity score 3-8;

** Nottingham eczema severity score 9-11.

*** Nottingham eczema severity score 12-15. Figures in parentheses are in percentage.

**Table 2:** The immunological parameters in control group versus subject group

Serum IL 4	Control group (*n =* 25)	Atopic dermatitis group (*n =* 25)
Range	10.83 – 30.51	47.27 – 82.26
Mean ± SD	21.51 ± 5.70	65.82 ± 8.58
P*		<0.001
Serum ECP		
Range	7.8 – 15.3	15.3 – 85.3
Mean ± SD	11.52 ± 2.26	36.97 ± 17.6
P*		<0.001
Serum total IgE		
Range	67.85 – 146.0	188.1 – 473.7
Mean ± SD	104.84 ± 25.84	281.25 ± 76.65
P*		<0.001

* *P* versus control group

## Discussion

The present study supported the association of R576 allele with atopy severity [Table 1] which has potential clinical applications. Early recognition of infants at risk for severe atopic disease by determination of their IL-4Rα 576 genotype followed by close medical follow-up and early environmental or pharmacologic intervention may delay, attenuate, or prevent the progression of disease. In adults identified as “at risk” for severe atopic diseases at the time of diagnosis, closer medical follow-up and early institution of therapy may alter their outcomes.

This result agreed with Deichmann *et al*,[18] who had confirmed the association of R576 allele with atopy, however, it has also found increased total IgE concentrations in patients with R576 allele and this agreed with Hershey *et al,*[19] who had reported an association between R576 allele and increased IgE level in AD and with Rosa-Rosa *et al*,[11] Beghe *et al,*[20] and Hytonen *et al*.[21] It seems that R576 gene might play a role in both conferring susceptibility to and modulating severity of atopy and/or asthma. Yandava *et al*,[22] had reported that the Q576R allele frequency was significantly higher in the asthmatics, the R576 allele was associated with increased serum total IgE.

Rogala *et al,*[23] hypothesized that there is strong evidence that the R576 allele might predispose to atopy in a Polish population, but their results did not reach statistical significance. Ober *et al*,[24] have found that R576 allele in the Hutterites and outbreed white, black, and Hispanic families showed evidence of association between variants in the IL4-Rα gene and atopy or asthma. Freidin *et al,*[25] have found comparable results in the population samples of Russians, Tajiks, Buryats, and Tuvinians racial, and ethnic specificity of this polymorphism was established.

The result regarding the association of R576 allele with atopy was in contradiction with Mitsuyasu *et al,*[26] who had found a similar R576 allele frequency in asthma and control Japanese groups. Patuzzo *et al,* could find no evidence of linkage or association of atopic asthma with R576 allele in Italian subjects. Whereas Haagerup *et al*,[27] had found that the R576 allele variant was not associated with five atopy phenotypes and concluded that the role of the R576 allele in the inheritance of atopy was insignificant in the Danish population.

The result regarding the association of R576 allele with atopy markers agreed with Cui *et al,*[28] who suggested that R576 genotypes confer genetic susceptibility to allergic asthma in Chinese and correlated with the increased plasma total IgE. Lee *et al*,[29] found similar results in Korean children, Isidoro-Garcia *et al*,[30] stated that there is an association between this allele and IgE levels in patients with positive skin-prick test and family history of atopy. These results were consistent with the results from earlier studies.[31,32]

In AD, there was a highly significant increase in serum ECP as compared to control group and there was a strong correlation between its level and disease severity. Similar results were obtained by Di Lorenzo *et al*, Kandil *et al*, Joseph-Bowen *et al,* and Higashi *et al.*[33–36] Eosinophils[37,38] contain abundant quantities of cationic granule proteins including major basic protein, ECP, eosinophil-derived neurotoxin, and eosinophil peroxidase. All can cause injurious microbial and tissue effects, and deposition has been demonstrated in AD lesions. These cells might be important effectors in AD. The prolonged survival of eosinophils in skin lesional sites is due to increased IL-5 expression by macrophages during the transition from acute to chronic AD and secretion of autocrine factors inhibiting eosinophil cell apoptosis.[39,40] Their role has been widely investigated in the cutaneous late-phase reaction (LPR) to allergens; studies in humans have demonstrated that systemic therapy with anti-IL-5 reduces eosinophilia. Studies indicate a possible role of eosinophils in connective tissue remodeling.

In AD group, there was also a highly significant increase in serum total IgE as compared with the control group and there was a highly significant positive correlation between increased serum total IgE levels and Nottingham eczema severity scoring system. These results indicate that serum total IgE may be used as an indicator of AD and its severity, especially in the peak season of allergy. Significant higher levels of serum IgE have been found in other studies.[41,42] Laske and Niggemann[42] have reported a significant correlation between dermatitis severity and serum total IgE levels. But, Wuthrich and Schmid-Grendelmeier[43] have reported that 45% of patients with AD have normal serum total IgE levels.

A majority of subjects identified as carrying a single copy of the mutant allele were found to have atopy, suggesting an intermediate dominant effect, with (increasing) homozygotes suffering more severely (gene dosage effect). However, the finding that some carriers of the R576 allele, including one who was homozygous, were not atopic, indicates that the penetrance of this allele may be modified by other factors. These may include distinct genetic loci that impart susceptibility to or protection from atopy, and environmental factors such as the level and duration of exposure to allergens.[44] This result conflicts with Zianil *et al*,[45] a French group, who reported that the Q576R allele was significantly more common among atopic subjects and seemed to act as a recessive.

The suggested molecular mechanism underlying the observed enhanced signaling with Q576R mutation and the association with R576 allele with atopy is that the substitution of arginine for glutamine at position 576 alters the binding profile of the adjacent phosphorylated tyrosine residue (Y575) and decreases the binding of phosphotyrosine phosphatases (SHP-1). SHP-1 dephosphorylates regulatory phosphotyrosine residues and has been implicated in the termination of IL-4 receptor signaling leading to exaggerated IL-4 responses. Hershey *et al*,[19] and Hanson *et al*,[46] stated that any alterations in the dephosphorylation of STAT-6 should have a potent effect on IL-4-mediated responses, of special interest R576 allele, supporting this explanation of the enhanced signaling with Q576R mutation and the association of R576 allele with atopy. This explanation coincides with the recommendation of Kamata *et al*,[47] who reported that reduced SHP-1 protein expression results in enhanced IL-4R-mediated signal transduction, Th2 cytokine production, and airway inflammation. Thus, SHP-1 could be a target signaling molecule for developing drugs for allergic asthma. However, Wang *et al,*[48] noted that R576 allele does not have a direct effect on IL-4 signal transduction. It is possible that multiple docking sites for such a phosphatase exist on the human IL-4Rα so that altering a single site would not result in a dramatic change in signaling. It should be emphasized that although decreased binding of SHP-1 to phosphorylated Y575 may provide explanation for the association of R576 allele with atopy, other mechanisms may be involved; these include alteration in the binding to phosphorylated Y575 of other, as yet unidentified, signaling intermediates, leading to exaggerated signaling as Kruse *et al*,[49] who suggested that phosphorylation of STAT-6 was increased if R576 allele is carried. The present study cannot rule out the presence in the IL-4R gene of additional mutations, not detected by this screening, that predispose persons to atopy either independently of or in synergy with the Q576R mutation as Risma *et al,*[50] reported that the association of R576 allele with atopic asthma was dependent on the coexistence of V75.

It could be postulated that patients with atopy having R576 allele may express a more highly active variant of the IL-4R; this mutation may predispose persons to allergic diseases by altering the signaling function of the receptor. So, R576 allele acts as an allergic susceptibility and disease-modifying gene and may serve as a clinically useful marker of asthma severity as one or two copies of R576 allele were associated with more severe disease. R576 allele correlates with markers of atopy, namely: IgE, ECP and eosinophil count.
